# Are there sex differences in the effect of type 2 diabetes in the incidence and outcomes of myocardial infarction? A matched-pair analysis using hospital discharge data

**DOI:** 10.1186/s12933-021-01273-y

**Published:** 2021-04-22

**Authors:** Ana Lopez-de-Andres, Rodrigo Jimenez-Garcia, Valentin Hernández-Barrera, Jose M. de Miguel-Yanes, Romana Albaladejo-Vicente, Rosa Villanueva-Orbaiz, David Carabantes-Alarcon, Jose J. Zamorano-Leon, Marta Lopez-Herranz, Javier de Miguel-Diez

**Affiliations:** 1 Department of Public Health & Maternal and Child Health, Faculty of Medicine, Universidad Complutense de Madrid, Madrid, Spain; 2 Preventive Medicine and Public Health Teaching and Research Unit, Health Sciences Faculty, Rey Juan Carlos University, Alcorcón, Madrid Spain; 3 Internal Medicine Department, Hospital General Universitario Gregorio Marañón, Universidad Complutense de Madrid, Instituto de Investigación Sanitaria Gregorio Marañón (IiSGM), Madrid, Spain; 4 Faculty of Nursing, Physiotherapy and Podology, Universidad Complutense de Madrid, 28040 Madrid, Spain; 5 Respiratory Care Department, Hospital General Universitario Gregorio Marañón, Universidad Complutense de Madrid, Instituto de Investigación Sanitaria Gregorio Marañón (IiSGM), Madrid, Spain

**Keywords:** Myocardial infarction, Type 2 diabetes mellitus, Sex differences, STEMI, NSTEMI, In-hospital mortality

## Abstract

**Background:**

To analyze incidence, use of therapeutic procedures, and in-hospital outcomes in patients with ST elevation myocardial infarction (STEMI) and non-ST elevation myocardial infarction (NSTEMI) according to the presence of type 2 diabetes (T2DM) in Spain (2016–2018) and to investigate sex differences.

**Methods:**

Using the Spanish National Hospital Discharge Database, we estimated the incidence of myocardial infarctions (MI) in men and women with and without T2DM aged ≥ 40 years. We analyzed comorbidity, procedures, and outcomes. We matched each man and woman with T2DM with a non-T2DM man and woman of identical age, MI code, and year of hospitalization. Propensity score matching was used to compare men and women with T2DM.

**Results:**

MI was coded in 109,759 men and 44,589 women (30.47% with T2DM). The adjusted incidence of STEMI (IRR 2.32; 95% CI 2.28–2.36) and NSTEMI (IRR 2.91; 95% CI 2.88–2.94) was higher in T2DM than non-T2DM patients, with higher IRRs for NSTEMI in both sexes. The incidence of STEMI and NSTEMI was higher in men with T2DM than in women with T2DM. After matching, percutaneous coronary intervention (PCI) was less frequent among T2DM men than non-T2DM men who had STEMI and NSTEMI. Women with T2DM and STEMI less frequently had a code for PCI that matched that of non-T2DM women. In-hospital mortality (IHM) was higher among T2DM women with STEMI and NSTEMI than in matched non-T2DM women. In men, IHM was higher only for NSTEMI. Propensity score matching showed higher use of PCI and coronary artery bypass graft and lower IHM among men with T2DM than women with T2DM for both STEMI and NSTEMI.

**Conclusions:**

T2DM is associated with a higher incidence of STEMI and NSTEMI in both sexes. Men with T2DM had higher incidence rates of STEMI and NSTEMI than women with T2DM. Having T2DM increased the risk of IHM after STEMI and NSTEMI among women and among men only for NSTEMI. PCI appears to be less frequently used in T2DM patients After STEMI and NSTEMI, women with T2DM less frequently undergo revascularization procedures and have a higher mortality risk than T2DM men.

**Supplementary Information:**

The online version contains supplementary material available at 10.1186/s12933-021-01273-y.

## Background

Diabetes is a major independent risk factor for cardiovascular disease (CVD) [1], particularly myocardial infarction (MI). The pattern of coronary artery disease in diabetic patients is often complex, with multiple lesions and widespread involvement [2], making it difficult to achieve complete revascularization and adversely affecting long-term prognosis [3, 4].

In Spain, the incidence of MI has been increasing over time in patients with type 2 diabetes mellitus (T2DM) [5]. Furthermore, T2DM is common in patients with MI, with a prevalence of around 20–30% [5, 6].

Several studies have also found a greater risk of death after MI in patients with diabetes than in those without diabetes [7, 8] in subtypes of coronary syndromes including unstable angina, ST-elevation MI (STEMI), and non-ST-elevation MI (NSTEMI) [9, 10].

Sex differences may play an active role in the incidence and outcomes of CVD, including MI [11]. Berger et al. [12] observed sex-based differences in their STEMI cohort, with 30-day mortality being higher among women (adjusted OR, 1.15; 95% CI 1.06–1.24), whereas mortality was lower among women in the NSTEMI cohort (adjusted OR, 0.77; 95% CI 0.63–0.95) and unstable angina cohort (adjusted OR, 0.55; 95% CI 0.43–0.70).

Traditional cardiovascular risk factors (CVRFs) such as diabetes are more associated with an excess relative risk in women than in men [11]. Using the UK Biobank to investigate the sex differences in risk factors for MI, Millet et al. [13] found that among women, diabetes was associated with higher hazard ratios for MI than in men: 2.91 (95% CI 1.56–5.45) for type 1 diabetes and 1.47 (95% CI 1.16–1.87) for T2DM.

CVRFs, such as high systolic and diastolic blood pressure and elevated lipid values (total cholesterol, LDL cholesterol, and triglycerides), worsen more rapidly in women after diagnosis of T2DM than in men [14]. Furthermore, a recent Spanish study of 32,638 T2DM patients performed over 20 years in primary care showed that CVRFs were more poorly controlled in women than in men [15].

Previous studies have found sex differences in incidence, use of revascularization procedures, and mortality in people with T2DM [16–19].

The increased risk of MI among men and women with diabetes, coupled with the increased incidence of T2DM worldwide in recent years, may substantially increase medical costs in the coming decades [20]. The abovementioned findings led us to compare the incidence, clinical characteristics, and in-hospital outcomes of patients admitted to Spanish hospitals from 2016 to 2018 with a primary diagnosis of MI according to the presence of T2DM and sex. We analyzed patients with STEMI and NSTEMI separately. Finally, we identified the variables associated with in-hospital mortality (IHM) for patients with T2DM according to sex and MI type.

## Methods

### Study design and data source

We conducted a retrospective observational study based on the Spanish National Hospital Discharge Database (SNHDD), which is managed by the Spanish Ministry of Health and includes over 95% of all hospital discharges in Spain resulting in data from more than 4.2 million discharges each year. The SNHDD has used the International Classification of Disease version 10 (ICD-10) for coding since the year 2016. The database provides up to 20 diagnoses and 20 procedures for each hospitalization. Detailed data on the SNHDD are available online [21].

### Study population

We analyzed data from all patients aged 40 years or over collected by the SNHDD in years 2016, 2017, and 2018.

Our study population includes patients discharged with a primary diagnosis of MI (STEMI and NSTEMI) using the specific ICD-10 codes shown in Additional file 1: Table S1. Patients with a code for “Subsequent STEMI and NSTEMI (I22.x)”, and “Other type of MI (I21.A.x)” were excluded. To control for the confounding effect of previous coronary disease, we excluded patients with an ICD-10 code for previous MI (I25.2).

The population was stratified according to sex and to the presence or absence of T2DM. A hospitalized patient was classified as having T2DM if a diagnosis code (ICD-10 E11.x) was recorded in any diagnosis field position (2–20) of the discharge report. The patient was excluded if a code for type 1 diabetes (E10.x) was found in any diagnosis field. In Spain, T2DM is defined as recommended by the American Diabetes Association [5, 22].

### Study variables

The primary outcome variables of interest were the incidence of STEMI and NSTEMI and in-hospital variables such as IHM and length of hospital stay (LOHS). Secondary outcomes of interest were use of coronary artery bypass graft (CABG) and percutaneous coronary intervention (PCI) during hospitalization.

Incidence rates were calculated using the Spanish population with and without T2DM estimated based on the Spanish National Health Survey 2017 and data provided by the Spanish National Statistics Institute, as reported by de Miguel-Yanes et al. [23].

Patient-level variables analyzed included age and sex. Comorbidity was assessed using the Charlson Comorbidity Index (CCI) based on the methods for ICD-10-coded administrative databases described by Sundararajan et al. [24].

A series of conditions and procedures were specifically identified and analyzed, as follows; obesity, hypertension, lipid metabolism disorders, kidney disease, atrial fibrillation, congestive heart failure, peripheral vascular disease, cerebrovascular disease, dementia, and mechanical ventilation (see Additional file 1: Table S1 and reference [24] for ICD10 codes).

### Matching method

Men and women with T2DM and MI were significantly older than non-diabetic patients, were unevenly distributed by type of MI, and differed significantly in the distribution of CVRFs and comorbid conditions (Additional file 2: Tables S2 and Additional file 3: Table S3). Therefore, and in order to control for these confounding factors and to make men and women with T2DM more comparable to those without diabetes, we matched, within the database of the SNHDD, a man with T2DM with a non-diabetic man with an identical age, type of MI (using all digits in the ICD 10 codes), and year of hospitalization. The same process was followed for women. If more than one man or woman without T2DM was available per man or woman with diabetes, selection was random.

### Statistical analysis

The statistical analysis was conducted separately for women and men.

Descriptive statistics were reported as mean with standard deviation or median with interquartile range for continuous variables and as frequency and percentage for categorical variables.

Incidence was analyzed using Poisson regression models adjusted for age and sex, when required, and providing incidence rate ratios (IRR) with 95% confidence intervals (95% CI) as a measure of association.

Continuous variables were compared using the *t*-test or Mann–Whitney test. Categorical variables were compared using the chi-square test.

Adjusted odds ratios (OR) were obtained using multivariable logistic regression to identify variables independently associated with IHM. Models were constructed separately for men and women and according to MI type. Finally, we analyzed the effect of sex using the entire database. Details on model construction have been provided elsewhere [23].

The matching process and statistical analysis were conducted using Stata version 14 (Stata, College Station, Texas, USA). Significance was set at a two-sided P-value of < 0.05.

### Sensitivity analyses

We complemented the logistic regression models with a propensity score matching (PSM) analysis to control for the confounding effect of baseline characteristics of men and women with T2DM. The PSM analysis was performed separately for the STEMI and NSTEMI cohorts and enabled us to obtain populations of T2DM men and T2DM women with a similar distribution of baseline variables that can affect the use of diagnostic procedures (PCI and CAGB) and hospital outcome variables (LOHS and IHM). The variables included in the PSM model were age, type of MI, and chronic conditions and CVRFs present on admission (Additional file 2: Table S2). The PSM approach has been described elsewhere [16].

### Ethical aspects

All investigators can request the databases of the SNHDD free of charge from the Spanish Ministry of Health [25]. The characteristics of this registry, which is anonymized and can be accessed through a specific request, makes it unnecessary to obtain approval by an ethics committee according to Spanish legislation.

## Results

A total of 154,348 hospital discharges (30.47% with T2DM) were recorded in Spain from 2016 to 2018 for patients aged ≥ 40 years with a primary diagnosis of MI. Men accounted for 71.11% (N = 109,759) and women 28.89% (N = 44,589). The overall prevalence of T2DM was higher among women than among men (34.13% vs. 28.97%; p < 0.001).

### Incidence of STEMI and NSTEMI according to T2DM

The total incidence of MI was higher (p < 0.001) among the T2DM population (536.91 per 100,000 persons with T2DM) than among those without T2DM (159.76 per 100,000 persons without T2DM) resulting in an adjusted IRR of 2.47 (95% CI 2.44–2.49). The IRR was 2.32 (95% CI 2.28–2.36) for STEMI and 2.91 (95% CI 2.88–2.94) for NSTEMI (Table 1).

**Table 1:** Incidence of myocardial infarction with and without ST elevation and according to presence of T2DM, sex and age groups

MI type	Age groups	Men			Women			Both		
		No T2DM	T2DM	p-value	No T2DM	T2DM	p-value	No T2DM	T2DM	p-value
		N (Inc/10^5^)	N (Inc/10^5^)		N (Inc/10^5^)	N (Inc/10^5^)		N (Inc/10^5^)	N (Inc/10^5^)	
STEMI	40–59 years	21,588 (107.51)	4438 (348.41)	< 0.001	3901 (18.89)	738 (86.95)	< 0.001	25,489 (62.57)	5176 (243.86)	< 0.001
	60–69 years	11,743 (203.89)	4332 (329.72)	< 0.001	3071 (45.96)	1126 (117.28)	< 0.001	14,814 (119.07)	5458 (240.03)	< 0.001
	70–79 years	8094 (223.4)	3933 (301.19)	< 0.001	3345 (75.45)	1976 (158.05)	< 0.001	11,439 (141.98)	5909 (231.17)	< 0.001
	≥ 80 years	6223 (283.03)	2816 (400.6)	< 0.001	5996 (159.99)	3176 (288.1)	< 0.001	12,219 (205.48)	5992 (331.9)	< 0.001
	All age groups	47,648 (150.5)	15,519 (337.63)	< 0.001	16,313 (45.93)	7016 (168.59)	< 0.001	63,961 (95.21)	22,535 (257.31)	< 0.001
NSTEMI	40–59 years	9734 (48.48)	2736 (214.79)	< 0.001	2268 (10.98)	564 (66.45)	< 0.001	12,002 (29.46)	3300 (155.47)	< 0.001
	60–69 years	7155 (124.23)	4372 (332.77)	< 0.001	2149 (32.16)	1243 (129.47)	< 0.001	9304 (74.79)	5615 (246.93)	< 0.001
	70–79 years	6796 (187.58)	4996 (382.59)	< 0.001	2970 (66.99)	2550 (203.96)	< 0.001	9766 (121.22)	7546 (295.22)	< 0.001
	≥ 80 years	6624 (301.26)	4179 (594.5)	< 0.001	5669 (151.26)	3847 (348.97)	< 0.001	12,293 (206.73)	8026 (444.57)	< 0.001
	All age groups	30,309 (95.73)	16,283 (354.26)	< 0.001	13,056 (36.76)	8204 (197.14)	< 0.001	43,365 (64.55)	24,487 (279.6)	< 0.001
Total	40–59 years,	31,322 (155.99)	7174 (563.21)	< 0.001	6169 (29.87)	1302 (153.4)	< 0.001	37,491 (92.04)	8476 (399.33)	< 0.001
	60–69 years	18,898 (328.13)	8704 (662.49)	< 0.001	5220 (78.13)	2369 (246.75)	< 0.001	24,118 (193.86)	11,073 (486.96)	< 0.001
	70–79 years	14,890 (410.98)	8929 (683.78)	< 0.001	6315 (142.44)	4526 (362.01)	< 0.001	21,205 (263.2)	13,455 (526.39)	< 0.001
	≥ 80 years	12,847 (584.29)	6995 (995.09)	< 0.001	11,665 (311.25)	7023 (637.07)	< 0.001	24,512 (412.21)	14,018 (776.47)	< 0.001
	All age groups	77,957 (246.23)	31,802 (691.89)	< 0.001	29,369 (82.69)	15,220 (365.74)	< 0.001	107,326 (159.76)	47,022 (536.91)	< 0.001

T2DM: Type 2 diabetes mellitus; Inc/10^5^: Incidence per 100,000 people with or without T2DM; STEMI: ST-elevation myocardial infarction; NSTEMI: non-ST elevation myocardial infarction

P values for comparison of the incidence between patients with and without T2DM using Poisson regression adjusted by age and sex when required

According to MI type by sex, we found that among men with T2DM, the incidence of STEMI was approximately twofold higher (337.63 vs. 150.5; IRR, 2.14; 95% CI 2.11–2.17) and that of NSTEMI was almost threefold higher (354.26 vs. 95.73; IRR, 2.86; 95% CI 2.81–2.91) than among men without T2DM.

Among T2DM women, the incidence of STEMI and NSTEMI was also significantly higher than among non-T2DM women [IRR, 2.47 (95% CI 2.44–2.49) and 3.26 (95% CI 3.24–3.29), respectively].

Men with T2DM had higher incidence rates of STEMI (337.63 vs. 168.59; p < 0.001) and NSTEMI (354.26 vs. 197.14; p < 0.001) than women with T2DM (Table 1). The corresponding adjusted IRR was 1.77 (95% CI 1.71–1.83) for STEMI and 1.59 (95% CI 1.52–1.67) for NSTEMI.

### Clinical characteristics and hospital outcomes for men and women with STEMI according to T2DM status

**Fig. 1:**
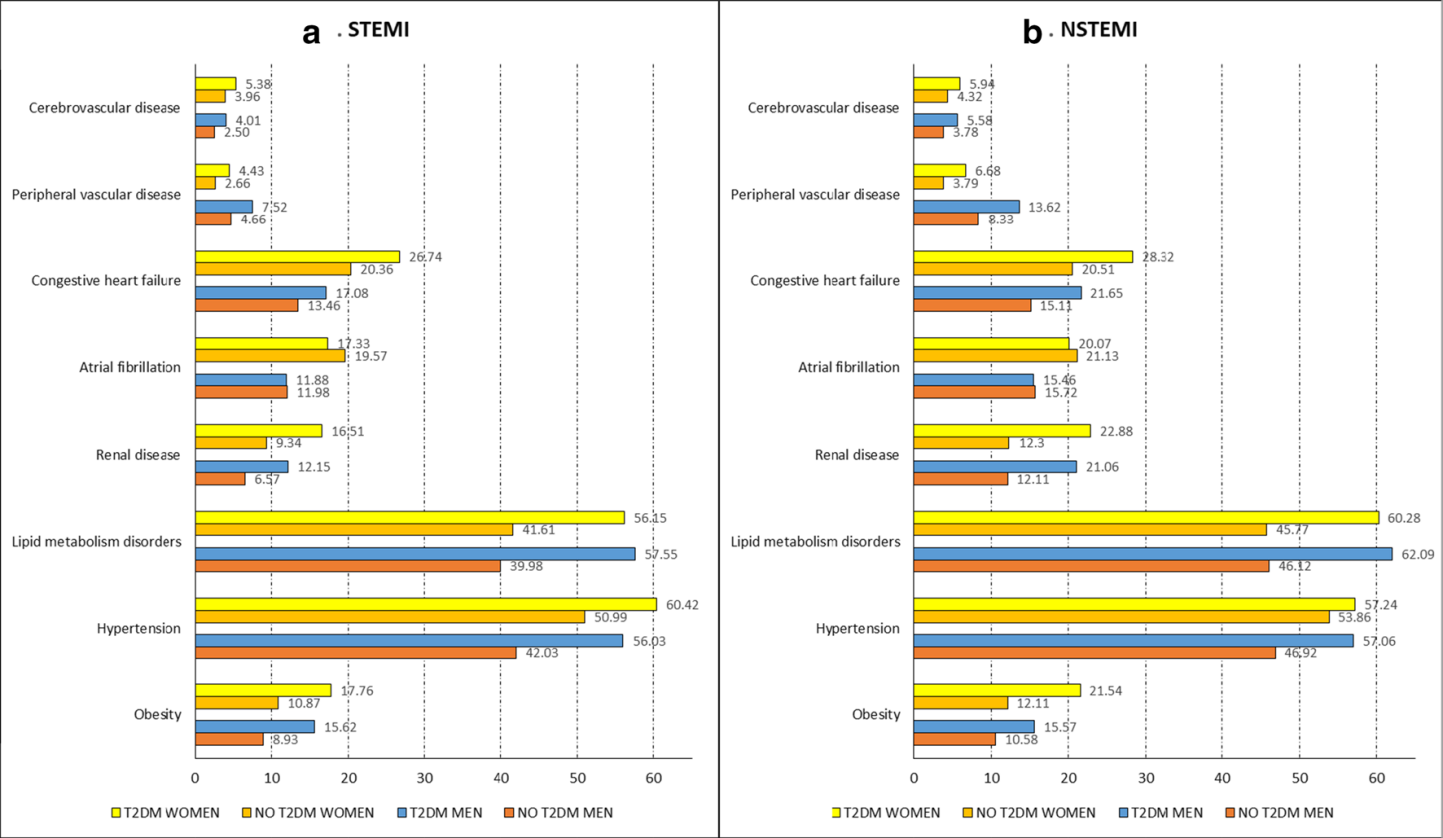
Prevalence of cardiovascular risk factors and clinical conditions according to sex, T2DM and MI type

The clinical characteristics, therapeutic procedures, and hospital outcomes after matching by age and MI type for men and women with STEMI according to the presence of T2DM are shown in Table 2 and Fig. 1a.

**Table 2:** Clinical characteristics, use of therapeutic procedures and hospital outcomes after matching by age and myocardial infarction type (ICD 10) for men and women hospitalized with STEMI according to the presence of T2DM

	Men			Women		
	No T2DM	T2DM	p-value	No T2DM	T2DM	p-value
STEMI involving left main coronary artery, n (%)	92 (0.59)	92 (0.59)	NA	28 (0.40)	28 (0.40)	NA
STEMI involving left anterior descending coronary artery, n (%)	1859 (11.99)	1859 (11.99)	NA	689 (9.85)	689 (9.85)	NA
STEMI involving other coronary artery of anterior wall, n (%)	3722 (24.01)	3722 (24.01)		1796 (25.68)	1796 (25.68)	NA
STEMI involving right coronary artery, n (%)	2163 (13.95)	2163 (13.95)	NA	771 (11.02)	771 (11.02)	NA
STEMI involving other coronary artery of inferior wall, n (%)	4204 (27.12)	4204 (27.12)	NA	1729 (24.72)	1729 (24.72)	NA
STEMI involving left circumflex coronary artery, n (%)	293 (1.89)	293 (1.89)	NA	91 (1.30)	91 (1.30)	NA
STEMI involving other sites, n (%)	843 (5.44)	843 (5.44)	NA	386 (5.52)	386 (5.52)	NA
STEMI of unspecified site, n (%)	2327 (15.01)	2327 (15.01)	NA	1504 (21.50)	1504 (21.50)	NA
Age, mean (SD)	67.33 (11.88)	67.33 (11.88)	NA	75.87 (11.43)	75.87 (11.43)	NA
CCI, mean (SD)	0.45 (0.74)	0.61 (0.56)	< 0.001	0.55 (0.47)	0.72 (0.65)	< 0.001
Obesity, n (%)	1384 (8.93)	2422 (15.62)	< 0.001	760 (10.87)	1242 (17.76)	< 0.001
Hypertension, n (%)	6516 (42.03)	8687 (56.03)	< 0.001	3566 (50.99)	4226 (60.42)	< 0.001
Lipid metabolism disorders, n (%)	6198 (39.98)	8922 (57.55)	< 0.001	2910 (41.61)	3927 (56.15)	< 0.001
Renal disease, n (%)	1019 (6.57)	1883 (12.15)	< 0.001	653 (9.34)	1155 (16.51)	< 0.001
Atrial fibrillation, n (%)	1857 (11.98)	1841 (11.88)	0.779	1369 (19.57)	1212 (17.33)	0.001
Congestive heart failure, n (%)	2087 (13.46)	2648 (17.08)	< 0.001	1424 (20.36)	1870 (26.74)	< 0.001
Peripheral vascular disease, n (%)	722 (4.66)	1166 (7.52)	< 0.001	186 (2.66)	310 (4.43)	< 0.001
Cerebrovascular disease, n (%)	387 (2.50)	621 (4.01)	< 0.001	277 (3.96)	376 (5.38)	< 0.001
Dementia, n (%)	170 (1.10)	215 (1.39)	0.021	272 (3.89)	334 (4.78)	0.010
Mechanical ventilation, n (%)	1075 (6.93)	1093 (7.05)	0.689	429 (6.13)	487 (6.96)	0.047
CABG, n (%)	180 (1.16)	235 (1.52)	0.007	44 (0.63)	52 (0.74)	0.413
PCI, n (%)	9629 (62.11)	9406 (60.67)	0.009	3384 (48.38)	3257 (46.57)	0.032
LOHS, median (IQR)	5.00 (5.00)	5.00 (5.00)	0.650	5.00 (5.00)	5.00 (5.00)	0.921
In-hospital mortality, n (%)	1184 (7.64)	1214 (7.83)	0.524	944 (13.50)	1091 (15.60)	< 0.001

*STEMI* ST-elevation myocardial infarction, *CCI* Charlson comorbidity index, *CABG* coronary artery bypass graft, *PCI* percutaneous coronary intervention, *LOHS* length of hospital stay, *NA* not applicable

The P values for the differences between patients with T2DM and No T2DM were calculated using Student’s t-test, or Mann–Whitney test or chi-square tests

Men with T2DM matched by age and STEMI type have more comorbid conditions than non-diabetic men. Remarkably higher prevalence values were recorded for obesity, hypertension, lipid metabolism disorders, kidney disease, congestive heart failure, peripheral vascular disease, and cerebrovascular disease.

The proportion of T2DM men who underwent CABG was small but significantly higher than matched non-diabetic men (1.52% vs. 1.16%: p = 0.007). On the other hand, non-diabetic men more frequently underwent PCI (62.11% vs. 60.67%; p = 0.009).

No significant differences were observed after matching for median LOHS (5 days for both groups) and IHM (7.83% for T2DM men and 7.64% for non-diabetic men).

When we compared women with and without T2DM who had had STEMI, the prevalence of most clinical conditions was significantly higher in women with T2DM than in matched non-diabetic women, as was the case with men (Table 2 and Fig. 1a). In contrast with men, the prevalence of atrial fibrillation was higher in women without diabetes (19.57% vs. 17.33%; p < 0.001).

Regarding procedures and hospital outcomes, we found that in women with T2DM, PCI was less frequent (46.57% vs. 48.38; p = 0.032) and IHM higher (15.60% vs. 13.50%; p < 0.001) than in matched nondiabetic women.

### Clinical characteristics and hospital outcomes for men and women with NSTEMI according to T2DM status

As can be seen in Table 3 and Fig. 1b, men with T2DM who had NSTEMI had more chronic conditions than men matched by age without T2DM. The same differences in the prevalence of clinical conditions found in men who had STEMI are found in the NSTEMI population. After NSTEMI, CABG was more frequent in diabetic men than in matched non-diabetic men (4.54% vs. 3.78%; p = 0.001); PCI was less frequent (42.88% vs. 46.38%; p < 0.001).

**Table 3:** Clinical characteristics, use of therapeutic procedures and hospital outcomes after matching by age and myocardial infarction type (ICD 10) for men and women hospitalized with NSTEMI according to the presence of T2DM

	Men			Woman		
	No T2DM	T2DM	p-value	No T2DM	T2DM	p-value
NSTEMI, n (%)	16,283	16,283	NA	8202	8202	NA
Age, mean (SD)	70.90 (11.05)	70.90 (11.05)	NA	76.93 (10.33)	76.93 (10.33)	NA
CCI, mean (SD)	0.61 (0.59)	0.86 (0.80)	< 0.001	0.60 (0.52)	0.83 (0.80)	< 0.001
Obesity, n (%)	1723 (10.58)	2535 (15.57)	< 0.001	993 (12.11)	1767 (21.54)	< 0.001
Hypertension, n (%)	7640 (46.92)	9291 (57.06)	< 0.001	441 (53.86)	4695 (57.24)	< 0.001
Lipid metabolism disorders, n (%)	7509 (46.12)	10,110 (62.09)	< 0.001	3754 (45.77)	4944 (60.28)	< 0.001
Renal disease, n (%)	1972 (12.11)	3430 (21.06)	< 0.001	1009 (12.30)	1877 (22.88)	< 0.001
Atrial fibrillation, n (%)	2559 (15.72)	2517 (15.46)	0.521	1724 (21.13)	1646 (20.07)	0.064
Congestive heart failure, n (%)	2460 (15.11)	3526 (21.65)	< 0.001	1682 (20.51)	2323 (28.32)	< 0.001
Peripheral vascular disease, n (%)	1357 (8.33)	2217 (13.62)	< 0.001	311 (3.79)	548 (6.68)	< 0.001
Cerebrovascular disease, n (%)	616 (3.78)	909 (5.58)	< 0.001	354 (4.32)	487 (5.94)	< 0.001
Dementia, n (%)	209 (1.28)	215 (1.32)	0.769	286 (3.49)	288 (3.51)	0.932
Mechanical ventilation, n (%)	586 (3.60)	829 (5.09)	< 0.001	270 (3.29)	389 (4.74)	< 0.001
CABG, n (%)	615 (3.78)	739 (4.54)	0.001	115 (1.40)	209 (2.55)	< 0.001
PCI, n (%)	7552 (46.38)	6982 (42.88)	< 0.001	2719 (33.15)	2715 (33.10)	0.947
LOHS, median (IQR)	5.00 (5.00)	6.00 (6.00)	< 0.001	6.00 (6.00)	6.00 (6.00)	0.89
In-hospital mortality, n (%)	706 (4.34)	790 (4.85)	0.026	523 (6.38)	601 (7.33)	0.016

*NSTEMI* non-ST elevation myocardial infarction, *CCI* Charlson comorbidity index, *CABG* coronary artery bypass graft, *PCI* percutaneous coronary intervention, *LOHS* length of hospital stay, *NA* not applicable

The P values for the differences between patients with T2DM and No T2DM were calculated using Student’s t-test, or Mann–Whitney test or chi-square tests

After matching, men with T2DM remained in hospital for a median of 6 days compared with 5 days for those without T2DM (p < 0.001). Moreover, IHM was significantly higher among T2DM men (4.85% vs. 4.34%; p = 0.026).

The prevalence of all the clinical conditions was significantly higher in women with T2DM than in matched nondiabetic women, except for dementia and atrial fibrillation, for which no significant differences were recorded (Table 3 and Fig. 1b).

Values for CABG (2.55% vs. 1.40%; p < 0.001) and IHM (7.33% vs. 6.38%; p = 0.016) were significantly higher among T2MD women who had NSTEMI than among matched women without diabetes.

### Multivariable analysis of IHM

The results of the multivariable logistic regression analysis are shown in Table 4. For men and women with and without T2DM, the risk of dying in hospital increased with age, kidney disease, congestive heart failure, peripheral vascular disease, cerebrovascular disease, dementia, and the need for mechanical ventilation during hospitalization. Atrial fibrillation was a risk factor only for men and women with T2DM.

**Table 4:** Multivariable logistic regression analysis of factors associated with in hospital mortality among patients with and without T2DM according to sex

	Men			Women		
	No T2DM	T2DM	Both	No T2DM	T2DM	Both
40–59 years	1	1	1	1	1	1
60–69 years	1.69 (1.36–2.11)	1.76 (1.43–2.16)	1.72 (1.48–2)	1.58 (1.02–2.45)	2.55 (1.57–4.14)	1.93 (1.4–2.65)
70–79 years	2.85 (2.31–3.5)	2.77 (2.27–3.37)	2.8 (2.43–3.23)	2.66 (1.8–3.93)	4.47 (2.83–7.05)	3.35 (2.5–4.49)
≥ 80 years	6.72 (5.47–8.26)	6.03 (4.94–7.35)	6.36 (5.51–7.34)	6.17 (4.21–9.03)	8.32 (5.31–13.05)	6.88 (5.16–9.18)
Obesity	0.64 (0.51–0.82)	0.79 (0.67–0.94)	0.74 (0.64–0.85)	0.78 (0.65–0.98)	0.79 (0.67–0.93)	0.78 (0.7–0.91)
Renal diseases	1.38 (1.19–1.6)	1.36 (1.27–1.55)	1.37 (1.24–1.51)	1.18 (1.10–1.43)	1.19 (1.03–1.37)	1.18 (1.06–1.32)
Atrial fibrillation	NS	1.16 (1.02–1.31)	1.13 (1.03–1.24)	NS	1.16 (1.02–1.32)	1.11 (1.01–1.21)
Congestive heart failure	1.99 (1.77–2.24)	1.64 (1.47–1.82)	1.79 (1.65–1.94)	1.72 (1.51–1.95)	1.43 (1.28–1.61)	1.55 (1.42–1.69)
Peripheral vascular disease	1.24 (1.03–1.49)	NS	1.17 (1.04–1.31)	1.52 (1.13–2.04)	NS	1.31 (1.09–1.57)
Cerebrovascular disease	1.42 (1.14–1.77)	1.72 (1.45–2.04)	1.59 (1.39–1.82)	1.67 (1.33–2.1)	1.55 (1.27–1.88)	1.6 (1.38–1.86)
Dementia	2.7 (2.07–3.52)	2.14 (1.65–2.78)	2.4 (2–2.89)	2.2 (1.77–2.72)	2.05 (1.68–2.49)	2.12 (1.84–2.45)
Mechanical ventilation	15.92 (13.94–18.19)	12.95 (11.43–14.67)	14.24 (13–15.59)	11.08 (9.22–13.32)	6.57 (5.56–7.77)	8.31 (7.34–9.39)
CABG	0.67 (0.48–0.93)	0.47 (0.34–0.65)	0.55 (0.44–0.7)	NS	0.53 (0.3–0.93)	0.66 (0.44–0.99)
PCI	0.38 (0.34–0.43)	0.44 (0.39–0.49)	0.41 (0.38–0.44)	0.37 (0.32–0.43)	0.37 (0.32–0.43)	0.37 (0.33–0.41)
STEMI/NSTEMI	2.42 (2.17–2.71)	2.33 (2.09–2.58)	2.37 (2.2–2.56)	2.74 (2.42–3.1)	2.81 (2.51–3.15)	2.78 (2.56–3.03)
T2DM	NA	NA	NS	NA	NA	1.14 (1.05–1.24)

Only variables with significant results in the multivariable regression are shown in the table

*STEMI* ST-elevation myocardial infarction, *NSTEMI* non-ST elevation myocardial infarction, *CABG* Coronary artery bypass graft, *PCI* percutaneous coronary intervention, *NS* not significant, *NA* not available

Obesity and undergoing PCI or CABG reduced IHM in all study groups, except for CABG among non-T2DM women.

Among diabetic men, the probability of dying was 2.33 times higher among those with STEMI than among those with NSTEMI (OR, 2.33; 95% CI 2.09–2.58). This risk was higher in women with T2DM (OR, 2.81; 95% CI 2.51–3.15).

After controlling for the study variables, having T2DM increased IHM after MI by 14% (OR, 1.14; 95% CI 1.05–1.24) (Table 4).

When we joined the database of men and women with T2DM and conducted a multivariable adjustment (Additional file 4: Table S4), we found that women with T2DM were significantly more likely to die in hospital than men with T2DM (OR, 1.24; 95% CI 1.15–1.35). In this final model, hypertension and lipid metabolism disorders were protective factors for IHM among patients with and without T2DM.

### Sensitivity analysis

Table 5 shows the distribution of the study variables for men and women with T2DM who had STEMI and NSTEMI after PSM. The differences in prevalence for most clinical characteristics lost significance after PSM, thus making the two populations more comparable. In the case of STEMI after PSM, frequency of use was significantly greater among men with T2DM than women with T2DM for PCI (54.35% vs. 46.56%; p < 0.001) and CABG (1.2% vs. 0.74%; p = 0.006).

**Table 5:** Clinical characteristics, use of therapeutic procedures and hospital outcomes after propensity score matching (PSM) for men and women with T2DM hospitalized with STEMI and NSTEMI

	STEMI			NSTEMI		
	T2DM Men	T2DM Women	p-value	T2DM Men	T2DM Women	p-value
STEMI involving left main coronary artery, n (%)	41 (0.58)	32 (0.46)	0.291	NA	NA	–
STEMI involving left anterior descending coronary artery, n (%)	768 (10.95)	689 (9.82)	0.029	NA	NA	–
STEMI involving other coronary artery of anterior wall, n (%)	1766 (25.17)	1796 (25.6)	0.554	NA	NA	–
STEMI involving right coronary artery, n (%)	859 (12.24)	771 (10.99)	0.021	NA	NA	–
STEMI involving other coronary artery of inferior wall, n (%)	1687 (24.05)	1729 (24.64)	0.403	NA	NA	–
STEMI involving left circumflex coronary artery, n (%)	96 (1.37)	94 (1.34)	0.885	NA	NA	–
STEMI involving other sites, n (%)	394 (5.62)	390 (5.56)	0.886	NA	NA	–
STEMI of unspecified site, n (%)	1405 (20.03)	1515 (21.59)	0.024	NA	NA	–
NSTEMI n (%)	NA	NA	–	8204 (100)	8204 (100)	NA
Age, mean (SD)	74.64 (10.77)	75.87 (11.43)	< 0.001	76.26 (9.90)	76.94 (10.33)	< 0.001
CCI, mean (SD)	0.74 (0.66)	0.72 (0.65)	0.191	0.87 (0.85)	0.83 (0.80)	0.011
Obesity, n (%)	1183 (16.86)	1246 (17.76)	0.160	1561 (19.03)	1767 (21.54)	< 0.001
Hypertension, n (%)	4273 (60.9)	4242 (60.46)	0.592	4699 (57.28)	4695 (57.23)	0.950
Lipid metabolism disorders, n (%)	3945 (56.23)	3939 (56.14)	0.919	4879 (59.47)	4944 (60.26)	0.301
Renal disease, n (%)	1127 (16.06)	1159 (16.52)	0.464	1902 (23.18)	1878 (22.89)	0.656
Atrial fibrillation, n (%)	1134 (16.16)	1217 (17.35)	0.061	1572 (19.16)	1647 (20.08)	0.140
Congestive heart failure, n (%)	1651 (23.53)	1875 (26.72)	< 0.001	2070 (25.23)	2324 (28.33)	< 0.001
Peripheral vascular disease, n (%)	320 (4.56)	310 (4.42)	0.684	491 (5.98)	548 (6.68)	0.068
Cerebrovascular disease, n (%)	347 (4.95)	376 (5.36)	0.268	420 (5.12)	487 (5.94)	0.022
Dementia, n (%)	204 (2.91)	334 (4.76)	< 0.001	187 (2.28)	288 (3.51)	< 0.001
Mechanical ventilation, n (%)	506 (7.21)	490 (6.98)	0.599	395 (4.81)	389 (4.74)	0.826
CABG, n (%)	84 (1.2)	52 (0.74)	0.006	266 (3.24)	209 (2.55)	0.008
PCI, n (%)	3813 (54.35)	3267 (46.56)	< 0.001	3222 (39.27)	2715 (33.09)	< 0.001
LOHS, median (IQR)	5 (5)	5.00 (5.00)	0.121	6 (6)	6.00 (6.00)	0.467
In-hospital mortality, n (%)	787 (11.22)	1095 (15.61)	< 0.001	529 (6.45)	602 (7.34)	0.025

*STEMI* ST-elevation myocardial infarction, *CCI* Charlson comorbidity index, *CABG* coronary artery bypass graft, *PCI* percutaneous coronary intervention, *LOHS* length of hospital stay, *NA* not applicable

The P values for the differences between patients with T2DM and No T2DM were calculated using Student’s t-test, or Mann–Whitney test or chi-square tests

Similar results were found in the NSTEMI cohort, with a higher proportion of men than women receiving PCI and CABG [39.27% vs. 33.09% (p = 0.008) and 3.24% vs. 2.55% (p = 0.008), respectively] Finally, IHM after PSM was higher among women than men who had STEMI (15.61% vs. 11.22%; p < 0.001) and NSTEMI (7.34% vs. 6.45%; p = 0.025). These results confirm those found in the multivariable logistic regression analysis (Additional file 4: Table S4).

## Discussion

This nationwide registry- and population-based observational study showed that incidence rates of STEMI and NSTEMI were higher in men and women with T2DM than in men and women without T2DM for all age groups analyzed. After pair-matching according to age, MI code, and year of hospitalization, use of PCI was lower in T2DM men and women with STEMI and in men with T2DM for NSTEMI. T2DM was associated with significantly higher IHM in women and men admitted to hospital for STEMI and only in men admitted for NSTEMI.

Women with T2DM hospitalized with STEMI or NSTEMI underwent revascularization procedures less frequently and had a higher risk of dying in hospital than T2DM men.

### Incidence of MI, revascularization procedures, and hospital mortality according to presence of T2DM

According to our database, patients with diabetes had a higher incidence of MI than those without diabetes. This finding has been constantly confirmed elsewhere [1, 5, 9, 13, 16, 17]. Furthermore, incidence rates of STEMI and NSTEMI were higher in T2DM men than in T2DM women. These trends are consistent with previous reports in people with diabetes and in the general population [26–28]. Despite the fact that the American Heart Association has analyzed MI in women in a separate document owing to the increasing weight of cardiovascular mortality in women [29], virtually every study shows persistently higher incidence rates of coronary disease in men [26–28].

The results of the present study indicate that during admission for STEMI or NSTEMI, men with T2DM undergo PCI less frequently than matched non-T2DM men. In addition, PCI was less frequently used during admission for STEMI in women with T2DM.

The underuse of revascularization procedures among people with diabetes has been reported for STEMI and NSTEMI in various countries [30–33]. One possible explanation is that PCI is technically more complicated owing to a higher frequency of diffuse and peripheral coronary artery disease among diabetic patients [34]. This is coherent with the more frequent use of CABG among patients with T2DM in our population.

Death rates during hospitalizations were higher in patients with diabetes than in those without diabetes, as described by Ahmed et al. [9], who obtained an adjusted OR of 1.07 (95% CI 1.05–1.08) for T2DM in patients who have had MI. Several studies have reported that, although the IHM of patients with MI decreased over time in both diabetic and non-diabetic patients, in-hospital deaths were always higher in diabetes patients [8, 9, 35, 36].

In the setting of MI, and in line with the explanation for the less frequent use of PCI, T2DM patients showed a greater propensity for multivessel stenosis involving several coronary arteries, as well as multiple stenoses in the same vessel, which can contribute to this higher IHM [8, 34].

Several cardiometabolic risk factors linked to T2DM have been found to be associated with increases in mortality after MI, including altered fasting glucose levels [37] and insulin treatment [38].

Furthermore, poorer outcomes in patients with diabetes may be explained by other factors such as vasculopathies secondary to hyperglycemia and hyperinsulinemia and coagulation system disorders involving platelets and the coagulation cascade [30].

### Sex differences in incidence, use of revascularization procedures, and IHM after MI among T2DM patients

The IRR obtained when we compared T2DM men and women with non-T2DM men and women for STEMI and NSTEMI was higher for women than for men (STEMI IRR 2.14 for men and 2.47 for women, NSTEMI IRR 2.86 for men and 3.26 for women), suggesting that T2DM may be a stronger relative risk factor for STEMI and NSTEMI in women than in men. This conclusion is consistent with most recent reviews and meta-analyses [16, 17], but not with all prior studies [39, 40]. These discrepancies may have arisen from differences in the level of adjustment for other CVRFs between the studies included. Peters et al. [16] analyzed 64 cohort studies and found a 44% greater excess risk ratio for incident coronary heart disease in women with diabetes than in their male counterparts.

Deterioration in CVRF levels among those with and without T2DM is greater in women than in men; therefore, women with diabetes are disadvantaged compared with men, even before their diagnosis [14, 17, 41]. Furthermore, the greater excess coronary risk associated with diabetes may reflect possible disparities in treatment, which favors men [17]. Additionally, in the UK, as in Spain, women with diabetes are less likely than men with diabetes to meet all recommended care requirements and might be less likely to achieve target values for treated CVRFs [15, 42]. However, in contrast to other types of CVD, one large meta-analysis did not find evidence that diabetes confers a greater excess risk for peripheral arterial disease in women than in men [43]. More research is needed to determine possible unknown mechanisms responsible for sex differences in diabetes-related cardiovascular risk, and this should also take into account hormonal factors [44]. Wang et al. [44] reported that sex hormones and diabetic vascular complications are sexually dimorphic, since CVD was associated with low total testosterone and high estradiol in men, while postmenopausal women present with high testosterone.

Before matching, but also once PSM was complete, we could see that use of PCI and CABG was more frequent among T2DM men than T2DM women for both STEMI and NSTEMI. This less invasive pattern in women has been reported elsewhere, and the possible reasons have been discussed by other investigators [18, 19, 28, 45, 46].

Lower revascularization rates can be explained, in part, by the higher frequency of alternative etiologies, such as Takotsubo (stress) cardiomyopathy, spontaneous coronary artery dissection, and coronary vasospasm in younger women with MI [18].

Another potential reason for lower use of revascularization is that women have more in-hospital complications after PCI than men and this may contribute to treatment decisions leading to a less invasive approach [47].

Walli-Attaei et al. [28] have suggested that the lower number of revascularization procedures observed in women might be partly explained by a lower burden of atherosclerosis in women. Across the spectrum of acute coronary syndrome, men have significantly more obstructive and multivessel disease than women [45, 46].

Regarding CAGB, the fact that women have smaller coronary arteries makes surgical grafting more challenging, thus potentially contributing to increased complications and mortality rates that could partly explain sex disparity in this procedure [45, 48, 49].

Finally, whether Spanish women and men differ in their priorities and preferences for revascularization therapies is unknown, and this could also contribute to the differences found. Future research should investigate whether women and men with diabetes differ in their suitability, contraindications, preferences, symptoms, anatomy, and access for revascularization procedures after MI.

The higher mortality in women than men with diabetes who experienced MI that we observed has been reported in meta-analyses [17, 50, 51].

Beside the differences in the course, prevalence, diagnosis, and treatment of CVRFs commented on above, other possible reasons for these sex differences have been posited. First, previous studies have suggested that women more frequently have MI with atypical symptoms than men, thus potentially contributing to later diagnosis and reduced provision of subsequent care [18]. Second, delays in receiving timely in‐hospital reperfusion therapy are due to longer times to calling medical services and longer times to attending the hospital for women [19, 28]. This is very important because women seem to be more vulnerable than men to prolonged untreated ischemia [19]. Third, the discrepancy between men and women in the prognosis of MI may result from differences in the frequency of coronary revascularization and invasive diagnostic procedures [18, 19, 28, 45, 46]. In our investigation, in addition to T2DM status, PCI was a strong predictor of survival for both sexes. And fourth, other potential mechanisms for sex-specific differences in mortality, as well as in incidence, may result from differences in biological factors [44].

### Factors associated with IHM among T2DM patients

In addition to sex and T2DM, we found that older age, kidney disease, atrial fibrillation, congestive heart failure, peripheral vascular disease, cerebrovascular disease, and dementia were risk factors for IHM. These predictors of worse outcome after MI have been reported elsewhere [5, 9, 18, 27, 33, 35, 49]. On the other hand, obesity, hypertension, and lipid metabolism disorders have been reported to protect against IHM [52, 53].

Interestingly, obesity reduced IHM for STEMI and NSTEMI in our investigation. The concept of an “*obesity survival paradox*” in patients with diabetes remains controversial [52–54]. While some studies have reported overweight and obesity as having an inverse relationship with increased mortality in normal-weight diabetic patients after MI [52], the results from the MONICA study indicated a survival benefit in obese post-MI patients, but only for those without diabetes. In obese patients with diabetes, reduced mortality was only present in those whose cardiovascular condition improved prior to MI [53].

In our investigation, atrial fibrillation was associated with higher IHM among men and women with T2DM. The negative effect of atrial fibrillation on the outcome of MI has previously been reported [5, 54]. T2DM, obesity, and atrial fibrillation frequently coexist, and relationships between them have not been fully elucidated [55]. Among patients with T2DM, the risk of incident atrial fibrillation is substantially increased for those with concomitant obesity or severe obesity [55]. However, there is an interaction between sex and body mass index (BMI), so that diabetic men have a higher risk of atrial fibrillation than diabetic women with equivalently elevated BMI [55].

Our multivariable analysis disclosed an unexpected protective effect of hypertension in mortality after MI. Bergmark et al. [56] analyzed data from 12,175 patients with T2DM and elevated cardiovascular risk and found that after multivariable adjustment, baseline systolic blood pressure (SBP) and diastolic blood pressure (DBP) had U-shaped relationships with major adverse cardiovascular events and cardiovascular death. The results of ONTARGET/TRANSCEND reached the same conclusions, with low levels of SBP (< 120 mmHg) and DBP (< 70 mmHg) being associated with increased cardiovascular outcomes (except stroke) and death among T2DM patients [57]. The physiological explanation hypothesized by other authors is that coronary filling is dependent on central aortic pressure, largely during diastole, and that this observation may explain why DBP is a cause of insufficient coronary perfusion, subclinical myocardial injury, and MI [56, 58]. In our opinion, patients without a code for hypertension in the SNHDD could have low levels of SBP or DBP, thus explaining this association.

Regarding lipid metabolism disorder, which was also a protective variable in our study, previous studies have shown that in patients who experienced STEMI and NSTEMI, hypercholesterolemia was associated with reduced mortality [59–61]. A common explanation for this phenomenon is inverse causation, which suggests that potentially serious disease conditions associated with low cholesterol level may contribute to increased mortality [59–62]. However, in a recent meta-analysis, after excluding terminal disease and mortality during the first year of observation, an initial low LDL-C level remained statistically associated with high mortality [62]. These findings may be explained by alterations in lipid synthesis, concentration, and composition in the in-hospital setting following MI due to an acute-phase reaction and inflammatory response [63].

Beside the coincidence with other investigations, the association between hypertension and lipid metabolism disorders found in our study must be interpreted with caution for a number of reasons. First, the SNHDD does not reflect current treatment patterns or provide measurements for blood pressure and blood lipids that would enable an in-depth analysis. Second, previous investigations have found that classic risk factors are frequently underreported in administrative databases, since risk factors per se do not influence reimbursement for acute hospital treatment of MI; therefore, the validity of these diagnoses is not high [64]. Furthermore, in our opinion, discharge reports provided by physicians tend to include more severe conditions in patients who died and more CVRFs among those who survived. However, this hypothesis must be confirmed in the future.

The risk of dying during the hospital stay was more than twofold higher for men and women with diabetes admitted for STEMI than for men and women with diabetes admitted for NSTEMI. This used to be the rule in older studies [65], although more recent reports show only slightly worse outcomes for STEMI [66], or even similar IHM rates for both conditions [67]. More invasive management of NSTEMI, which shares the presence of myocardial necrosis with STEMI, probably explains the closer rates found in more recent research work.

### Strengths and limitations

The strengths of our study lie in the large sample size [with data from over 154,348 episodes of MI (30.47% with T2DM)], the fact that it covers the population of an entire country (> 95% of all hospital admissions), the standardized methodology, which has been extensively used for research in Spain [5, 23, 68–70], and the reliable coding of acute coronary syndrome in the SNHDD [68]. Nevertheless, our findings are also subject to several limitations. Our data source is an administrative database that is supported by the information that physicians recorded in the discharge report, which also depends on manual coding by administrative staff. Despite a pair-matching process and PSM, which helped to attenuate differences in baseline characteristics and clinical variables, residual confounding cannot be completely eliminated in observational studies. In addition, anonymity precludes the extraction of specific data (i.e., people who moved from one hospital to another could appear twice).

The validity of the diabetes diagnosis in the SNHDD has been evaluated in two previous Spanish studies, which reported sensitivity of 55% and 63.7%, specificity of around 97% and a kappa concordance index of 0.6 and 0.7 [69, 70]. The fact that sensitivity is not very high implies that the condition is not coded in some patients who really have T2DM (false negatives), whereas the very high specificity means that almost all patients with a T2DM code really had this disease (very few false positives). To our knowledge, the validity of the diabetes diagnosis does not differ between men and women; therefore, in our opinion, the misclassification bias would be nondifferential and would not affect the conclusions of our investigation.

Finally, the SNHDD does not include duration and treatment of T2DM or other CVRFs and comorbid conditions that have a major impact on the risk of mortality and cardiovascular events.

## Conclusions

In summary, the incidence of STEMI and NSTEMI was higher in men and women with T2DM than in those without diabetes, with the incidence being higher in T2DM men than in T2DM women. Women with T2DM undergo fewer revascularization procedures and have higher mortality risk than T2DM men after STEMI and NSTEMI.

Our findings should be taken into consideration when planning future actions to improve the treatment and care these patients receive. Research efforts should focus on eliminating these sex-related disparities in our health system.

## Supplementary Information

**Additional file 1: Table S1.** International Classification of Disease 10th edition (ICD-10) codes for the clinical diagnosis and procedures used in this investigation.

**Additional file 2: Table S2.** Clinical characteristics, use of therapeutic procedures and hospital outcomes before matching for men and women patients with STEMI according to T2DM status.

**Additional file 3: Table S3.** Clinical characteristics, use of therapeutic procedures and hospital outcomes before matching for men and women patients with NSTEMI according to T2DM status.

**Additional file 4: Table S4.** Logistic regression factors associated with IHM after myocardial infarction among all patients and according to the presence of T2DM to assess the sex differences.

## Data Availability

According to the contract signed with the Spanish Ministry of Health and Social Services, which provided access to the databases from the Spanish National Hospital Database (*Conjunto Mínimo Basico de Datos*; CMBD), we cannot share the databases with any other investigator, and we have to destroy the databases once the investigation has concluded. Consequently, we cannot upload the databases to any public repository. However, any investigator can apply for access to the databases by filling out the questionnaire available at http://www.msssi.gob.es/estadEstudios/estadisticas/estadisticas/estMinisterio/SolicitudCMBDdocs/Formulario_Peticion_Datos_CMBD.pdf. All other relevant data are included in the paper.

## Abbreviations

CABG: Coronary artery bypass graft
CHD: Coronary heart disease
CVD: Cardiovascular disease
CVRFs: Cardiovascular risk factors
CCI: Charlson Comorbidity Index
CAD: Coronary artery disease
DBP: Diastolic blood pressure
IHM: In hospital mortality
IRR: Incidence rate ratios
LOHS: Length of hospital stay
MI: Myocardial infarction
NSTEMI: Non-ST-elevation MI
OR: Odds ratio
PCI: Percutaneous coronary intervention
PSM: Propensity score matching
SBP: Systolic blood pressure
SNHDD: Spanish National Hospital Discharge Database
STEMI: ST-elevation MI
T1DM: Type 1 diabetes mellitus
T2DM: Type 2 diabetes mellitus

## References

[1] Rawshani A, Rawshani A, Franzén S, Eliasson B, Svensson AM, Miftaraj M (2017). Mortality and cardiovascular disease in type 1 and type 2 diabetes. N Engl J Med.

[2] Nicholls SJ, Tuzcu EM, Kalidindi S, Wolski K, Moon KW, Sipahi I (2008). Effect of diabetes on progression of coronary atherosclerosis and arterial remodeling: a pooled analysis of 5 intravascular ultrasound trials. J Am Coll Cardiol.

[3] Flaherty JD, Davidson CJ (2005). Diabetes and coronary revascularization. JAMA.

[4] Marcheix B, Eynden FV, Demers P, Bouchard D, Cartier R (2008). Influence of diabetes mellitus on long-term survival in systematic off-pump coronary artery bypass surgery. Ann Thorac Surg.

[5] Lopez-de-Andres A, Jimenez-Garcia R, Hernandez-Barrera V, Jimenez-Trujillo I, Gallardo-Pino C, de Miguel AG (2014). National trends over one decade in hospitalization for acute myocardial infarction among Spanish adults with type 2 diabetes: cumulative incidence, outcomes and use of percutaneous coronary intervention. PLoS ONE.

[6] Cimci M, Witassek F, Radovanovic D, Rickli H, Pedrazzini GB, Erne P (2020). Temporal trends in cardiovascular risk factors prevalence in patients with myocardial infarction. Eur J Clin Invest.

[7] Zinman B, Wanner C, Lachin JM, Fitchett D, Bluhmki E, Hantel S (2015). Empagliflozin, cardiovascular outcomes, and mortality in type 2 diabetes. N Engl J Med.

[8] Schmitt VH, Hobohm L, Münzel T, Wenzel P, Gori T, Keller K (2020). Impact of diabetes mellitus on mortality rates and outcomes in myocardial infarction. Diabetes Metab.

[9] Ahmed B, Davis HT, Laskey WK (2014). In-hospital mortality among patients with type 2 diabetes mellitus and acute myocardial infarction: results from the national inpatient sample, 2000–2010. J Am Heart Assoc.

[10] Marfella R, Sardu C, Balestrieri ML, Siniscalchi M, Minicucci F, Signoriello G (2018). Effects of incretin treatment on cardiovascular outcomes in diabetic STEMI-patients with culprit obstructive and multivessel non obstructive-coronary-stenosis. Diabetol Metab Syndr.

[11] Stehli J, Duffy SJ, Burgess S, Kuhn L, Gulati M, Chow C (2021). Sex disparities in myocardial infarction: biology or bias?. Heart Lung Circ.

[12] Berger JS, Elliott L, Gallup D, Roe M, Granger CB, Armstrong PW (2009). Sex differences in mortality following acute coronary syndromes. JAMA.

[13] Millett ERC, Peters SAE, Woodward M (2018). Sex differences in risk factors for myocardial infarction: cohort study of UK Biobank participants. BMJ.

[14] Bancks MP, Akhabue E, Rana JS, Reis JP, Schreiner PJ, Yano Y (2020). Sex differences in cardiovascular risk factors before and after the development of type 2 diabetes and risk for incident cardiovascular disease. Diabetes Res Clin Pract.

[15] Galbete A, Cambra K, Forga L, Baquedano FJ, Aizpuru F, Lecea O (2019). Achievement of cardiovascular risk factor targets according to sex and previous history of cardiovascular disease in type 2 diabetes: a population-based study. J Diabetes Complicat.

[16] Peters SA, Huxley RR, Woodward M (2014). Diabetes as risk factor for incident coronary heart disease in women compared with men: a systematic review and meta-analysis of 64 cohorts including 858,507 individuals and 28,203 coronary events. Diabetologia.

[17] Dong X, Cai R, Sun J, Huang R, Wang P, Sun H, Tian S, Wang S (2017). Diabetes as a risk factor for acute coronary syndrome in women compared with men: a meta-analysis, including 10 856 279 individuals and 106 703 acute coronary syndrome events. Diabetes Metab Res Rev.

[18] Khera S, Kolte D, Gupta T, Subramanian KS, Khanna N, Aronow WS (2015). Temporal trends and sex differences in revascularization and outcomes of ST-segment elevation myocardial infarction in younger adults in the United States. J Am Coll Cardiol.

[19] Bugiardini R, Ricci B, Cenko E, Vasiljevic Z, Kedev S, Davidovic G (2017). Delayed care and mortality among women and men with myocardial infarction. J Am Heart Assoc.

[20] Mauvais-Jarvis F, Bairey Merz N, Barnes PJ, Brinton RD, Carrero JJ, DeMeo DL (2020). Sex and gender: modifiers of health, disease, and medicine. Lancet.

[21] Ministerio de Sanidad, Servicios Sociales e Igualdad. Real Decreto 69/2015, de 6 de febrero, por el que se regula el Registro de Actividad de Atención Sanitaria Especializada. (Spanish National Hospital Discharge Database) BOE. 2015;35:10789–809. https://www.mscbs.gob.es/estadEstudios/estadisticas/docs/BOE_RD_69_2015_RAE_CMBD.pdf. Accessed 12 Nov 2020.

[22] Rojo-Martínez G, Valdés S, Soriguer F, Vendrell J, Urrutia I, Pérez V, Ortega E (2020). Incidence of diabetes mellitus in Spain as results of the nation-wide cohort di@bet.es study. Sci Rep.

[23] Miguel-Yanes JM, Jiménez-García R, Hernández-Barrera V, de Miguel-Díez J, Méndez-Bailón M, Muñoz-Rivas N (2019). Infective endocarditis according to type 2 diabetes mellitus status: an observational study in Spain, 2001–2015. Cardiovasc Diabetol.

[24] Sundararajan V, Henderson T, Perry C, Muggivan A, Quan H, Ghali WA (2004). New ICD-10 version of the Charlson comorbidity index predicted in-hospital mortality. J Clin Epidemiol.

[25] Ministerio de Sanidad, Consumo y Bienestar Social. Solicitud de extracción de datos—extraction request (Spanish National Hospital Discharge Database). https://www.mscbs.gob.es/estadEstudios/estadisticas/estadisticas/estMinisterio/SolicitudCMBDdocs/2018_Formulario_Peticion_Datos_RAE_CMBD.pdf. Accessed 12 Nov 2020.

[26] Norhammar A, Stenestrand U, Lindbäck J, Wallentin L, Register of Information and Knowledge about Swedish Heart Intensive Care Admission (RIKS-HIA) (2008). Women younger than 65 years with diabetes mellitus are a high-risk group after myocardial infarction: a report from the Swedish Register of Information and Knowledge about Swedish Heart Intensive Care Admission (RIKS-HIA). Heart.

[27] Maier B, Thimme W, Kallischnigg G, Graf-Bothe C, Röhnisch JU, Hegenbarth C (2006). Does diabetes mellitus explain the higher hospital mortality of women with acute myocardial infarction? Results from the Berlin myocardial infarction registry. J Investig Med.

[28] Walli-Attaei M, Joseph P, Rosengren A, Chow CK, Rangarajan S, Lear SA (2020). Variations between women and men in risk factors, treatments, cardiovascular disease incidence, and death in 27 high-income, middle-income, and low-income countries (PURE): a prospective cohort study. Lancet.

[29] Mehta LS, Beckie TM, DeVon HA, Grines CL, Krumholz HM, Johnson MN (2016). Acute myocardial infarction in women: a scientific statement from the American Heart Association. Circulation.

[30] Rousan TA, Pappy RM, Chen AY, Roe MT, Saucedo JF (2014). Impact of diabetes mellitus on clinical characteristics, management, and in-hospital outcomes in patients with acute myocardial infarction (from the NCDR). Am J Cardiol.

[31] Elbarouni B, Ismaeil N, Yan RT, Fox KA, Connelly KA, Baer C (2011). Temporal changes in the management and outcome of Canadian diabetic patients hospitalized for non-ST-elevation acute coronary syndromes. Am Heart J.

[32] Gustafsson I, Hvelplund A, Hansen KW, Galatius S, Madsen M, Jensen JS (2015). Underuse of an invasive strategy for patients with diabetes with acute coronary syndrome: a nationwide study. Open Heart.

[33] Chakraborty S, Amgai B, Bandyopadhyay D, Patel N, Hajra A, Narasimhan B (2021). Acute myocardial infarction in the young with diabetes mellitus—national inpatient sample study with sex-based difference in outcomes. Int J Cardiol.

[34] Goraya TY, Leibson CL, Palumbo PJ, Weston SA, Killian JM, Pfeifer EA (2002). Coronary atherosclerosis in diabetes mellitus: a population-based autopsy study. J Am Coll Cardiol.

[35] Read SH, Fischbacher CM, Colhoun HM, Gasevic D, Kerssens JJ, McAllister DA (2019). Trends in incidence and case fatality of acute myocardial infarction, angina and coronary revascularisation in people with and without type 2 diabetes in Scotland between 2006 and 2015. Diabetologia.

[36] Bauters C, Lemesle G, de Groote P, Lamblin N (2016). A systematic review and meta-regression of temporal trends in the excess mortality associated with diabetes mellitus after myocardial infarction. Int J Cardiol.

[37] Liang H, Guo YC, Chen LM, Li M, Han WZ, Zhang X (2016). Relationship between fasting glucose levels and in-hospital mortality in Chinese patients with acute myocardial infarction and diabetes mellitus: a retrospective cohort study. BMC Cardiovasc Disord.

[38] Hoebers LP, Claessen BE, Woudstra P, DeVries JH, Wykrzykowska JJ, Vis MM (2014). Long-term mortality after primary percutaneous coronary intervention for ST-segment elevation myocardial infarction in patients with insulin-treated versus non-insulin-treated diabetes mellitus. EuroIntervention.

[39] Kanaya AM, Grady D, Barrett-Connor E (2002). Explaining the sex difference in coronary heart disease mortality among patients with type 2 diabetes mellitus: a meta-analysis. Arch Intern Med.

[40] Hyvärinen M, Tuomilehto J, Laatikainen T, Söderberg S, Eliasson M, Nilsson P (2009). The impact of diabetes on coronary heart disease differs from that on ischaemic stroke with regard to the gender. Cardiovasc Diabetol.

[41] Wannamethee SG, Papacosta O, Lawlor DA, Whincup PH, Lowe GD, Ebrahim S (2012). Do women exhibit greater differences in established and novel risk factors between diabetes and non-diabetes than men? The British regional heart study and British women’s heart health study. Diabetologia.

[42] Franzini L, Ardigò D, Cavalot F, Miccoli R, Rivellese AA, Trovati M (2013). Women show worse control of type 2 diabetes and cardiovascular disease risk factors than men: results from the MIND.IT Study Group of the Italian Society of Diabetology. Nutr Metab Cardiovasc Dis.

[43] Chase-Vilchez AZ, Chan IHY, Peters SAE, Woodward M (2020). Diabetes as a risk factor for incident peripheral arterial disease in women compared to men: a systematic review and meta-analysis. Cardiovasc Diabetol.

[44] Wang C, Zhang W, Wang Y, Wan H, Chen Y, Xia F (2019). Novel associations between sex hormones and diabetic vascular complications in men and postmenopausal women: a cross-sectional study. Cardiovasc Diabetol.

[45] Gudnadottir GS, Andersen K, Thrainsdottir S, James SK, Lagerqvist B, Gudnason T (2017). Gender differences in coronary angiography, subsequent interventions, and outcomes among patients with acute coronary syndromes. Am Heart J.

[46] Hansen KW, Soerensen R, Madsen M, Madsen JK, Jensen JS, von Kappelgaard LM (2015). Developments in the invasive diagnostic—therapeutic cascade of women and men with acute coronary syndromes from 2005 to 2011: a nationwide cohort study. BMJ Open.

[47] Duvernoy CS, Smith DE, Manohar P, Schaefer A, Kline-Rogers E, Share D (2010). Gender differences in adverse outcomes after contemporary percutaneous coronary intervention: an analysis from the Blue Cross Blue Shield of Michigan Cardiovascular Consortium (BMC2) percutaneous coronary intervention registry. Am Heart J.

[48] Ahmed B, Lischke S, Holterman LA, Straight F, Dauerman HL (2010). Angiographic predictors of vascular complications among women undergoing cardiac catheterization and intervention. J Invasive Cardiol.

[49] Swaminathan RV, Feldman DN, Pashun RA, Patil RK, Shah T, Geleris JD (2016). Gender differences in in-hospital outcomes after coronary artery bypass grafting. Am J Cardiol.

[50] Huxley R, Barzi F, Woodward M (2006). Excess risk of fatal coronary heart disease associated with diabetes in men and women: meta-analysis of 37 prospective cohort studies. BMJ.

[51] Wang Y, O'Neil A, Jiao Y, Wang L, Huang J, Lan Y (2019). Sex differences in the association between diabetes and risk of cardiovascular disease, cancer, and all-cause and cause-specific mortality: a systematic review and meta-analysis of 5,162,654 participants. BMC Med.

[52] Wang L, Liu W, He X, Chen Y, Lu J, Liu K (2016). Association of overweight and obesity with patient mortality after acute myocardial infarction: a meta-analysis of prospective studies. Int J Obes.

[53] Colombo MG, Meisinger C, Amann U, Heier M, von Scheidt W, Kuch B (2015). Association of obesity and long-term mortality in patients with acute myocardial infarction with and without diabetes mellitus: results from the MONICA/KORA myocardial infarction registry. Cardiovasc Diabetol.

[54] Jabre P, Roger VL, Murad MH, Chamberlain AM, Prokop L, Adnet F (2011). Mortality associated with atrial fibrillation in patients with myocardial infarction: a systematic review and meta-analysis. Circulation.

[55] Singleton MJ, German CA, Soliman EZ, Whalen SP, Bhave PD, Bertoni AG (2020). Body mass index, sex, and incident atrial fibrillation in diabetes: the ACCORD trial. JACC Clin Electrophysiol.

[56] Bergmark BA, Scirica BM, Steg PG, Fanola CL, Gurmu Y, Mosenzon O (2018). Blood pressure and cardiovascular outcomes in patients with diabetes and high cardiovascular risk. Eur Heart J.

[57] Böhm M, Schumacher H, Teo KK, Lonn EM, Mahfoud F, Mann JFE (2019). Cardiovascular outcomes and achieved blood pressure in patients with and without diabetes at high cardiovascular risk. Eur Heart J.

[58] McEvoy JW, Chen Y, Rawlings A, Hoogeveen RC, Ballantyne CM, Blumenthal RS (2016). Diastolic blood pressure, subclinical myocardial damage, and cardiac events: implications for blood pressure control. J Am Coll Cardiol.

[59] Cho KH, Jeong MH, Ahn Y, Kim YJ, Chae SC, Hong TJ (2010). Low-density lipoprotein cholesterol level in patients with acute myocardial infarction having percutaneous coronary intervention (the cholesterol paradox). Am J Cardiol.

[60] Nozue T (2016). Low-density lipoprotein cholesterol level and statin therapy in patients with acute myocardial infarction (cholesterol paradox). Circ J.

[61] Reddy VS, Bui QT, Jacobs JR, Begelman SM, Miller DP, French WJ (2015). Relationship between serum low-density lipoprotein cholesterol and in-hospital mortality following acute myocardial infarction (the lipid paradox). Am J Cardiol.

[62] Ravnskov U, Diamond DM, Hama R, Hamazaki T, Hammarskjöld B, Hynes N (2016). Lack of an association or an inverse association between low-density-lipoprotein cholesterol and mortality in the elderly: a systematic review. BMJ Open.

[63] Pitt B, Loscalzo J, Ycas J, Raichlen JS (2008). Lipid levels after acute coronary syndromes. J Am Coll Cardiol.

[64] Maier B, Wagner K, Behrens S, Bruch L, Busse R, Schmidt D, Schühlen H (2016). Comparing routine administrative data with registry data for assessing quality of hospital care in patients with myocardial infarction using deterministic record linkage. BMC Health Serv Res.

[65] Hasdai D, Behar S, Wallentin L, Danchin N, Gitt AK, Boersma E (2002). A prospective survey of the characteristics, treatments and outcomes of patients with acute coronary syndromes in Europe and the Mediterranean basin; the Euro heart survey of acute coronary syndromes (Euro heart survey ACS). Eur Heart J.

[66] Komiyama K, Nakamura M, Tanabe K, Niikura H, Fujimoto H, Oikawa K (2018). In-hospital mortality analysis of Japanese patients with acute coronary syndrome using the Tokyo CCU network database: applicability of the GRACE risk score. J Cardiol.

[67] Montalescot G, Dallongeville J, Van Belle E, Rouanet S, Baulac C, Degrandsart A (2007). STEMI and NSTEMI: are they so different? 1 year outcomes in acute myocardial infarction as defined by the ESC/ACC definition (the OPERA registry). Eur Heart J.

[68] Bernal JL, Barrabés JA, Íñiguez A, Fernández-Ortiz A, Fernández-Pérez C, Bardají A (2019). Clinical and administrative data on the research of acute coronary syndrome in Spain. Minimum basic data set validity. Rev Esp Cardiol.

[69] Ribera A, Marsal JR, Ferreira-González I, Cascant P, Cascant P, Pons JM, Mitjavila F (2008). Predicting in-hospital mortality with coronary bypass surgery using hospital discharge data: comparison with a prospective observational study. Rev Esp Cardiol.

[70] Rodrigo-Rincón I, Martin-Vizcaíno MP, Tirapu-León B, Zabalza-López P, Abad-Vicente FJ, Merino-Peralta A (2016). Usefulness of administrative databases for risk adjustment of adverse events in surgical patients. Cir Esp.

