# Supplementary material for: Are there sex differences in the effect of type 2 diabetes in the incidence and outcomes of myocardial infarction? A matched-pair analysis using hospital discharge data
Source: Cardiovasc Diabetol. 2021 Apr 22;20:81. doi: 10.1186/s12933-021-01273-y (PMC8063379; doi:10.1186/s12933-021-01273-y)
Supplement: Supplementary file 1 — Additional file 1: Table S1. International Classification of Disease 10th edition (ICD-10) codes for the clinical diagnosis and procedures used in this investigation. [file 12933_2021_1273_MOESM1_ESM.docx]

TABLE S1. International Classification of Disease 10^th^ edition (ICD-10) codes for the clinical diagnosis and procedures used in this investigation.

| Clinical diagnosis and procedures | ICD-10 codes |
| --- | --- |
| STEMI involving left main coronary artery | I21.01 |
| STEMI involving left anterior descending coronary artery | I21.02 |
| STEMI involving other coronary artery of anterior wall | I21.09 |
| STEMI involving right coronary artery | I21.11 |
| STEMI involving other coronary artery of inferior wall | I21.19 |
| STEMI involving left circumflex coronary artery | I21.21 |
| STEMI involving other sites | I21.29 |
| STEMI of unspecified site | I21.3 |
| NSTEMI | I21.4 |
| Obesity | E66.X |
| Hypertension | I10, I16.6 |
| Lipid metabolism disorders | E78.0X-E78.5 |
| Atrial fibrillation | I48.0, I48.1, I48.2, I48.91 |
| Mechanical ventilation | 5A1945Z, 5A1955Z, 5A1935Z,5A09357, 5A09457, 5A09557 |
| CABG | 02100XX, 02110XX, 02120XX, 02130XX |
| PCI | 02703XX, 02713XX, 02723XX, 02733XX |

STEMI; ST-elevation myocardial infarction. NSTEMI; non-ST elevation myocardial infarction CABG: Coronary artery bypass graft; PCI: Percutaneous coronary intervention.
