# Supplementary material for: Are there sex differences in the effect of type 2 diabetes in the incidence and outcomes of myocardial infarction? A matched-pair analysis using hospital discharge data
Source: Cardiovasc Diabetol. 2021 Apr 22;20:81. doi: 10.1186/s12933-021-01273-y (PMC8063379; doi:10.1186/s12933-021-01273-y)
Supplement: Supplementary file 2 — Additional file 2: Table S2. Clinical characteristics, use of therapeutic procedures and hospital outcomes before matching for men and women patients with STEMI according to T2DM status. [file 12933_2021_1273_MOESM2_ESM.docx]

TABLE S2. Clinical characteristics, use of therapeutic procedures and hospital outcomes before matching for men and women patients with STEMI according to T2DM status.

|  | **MEN** | | | **WOMEN** | | |
| --- | --- | --- | --- | --- | --- | --- |
|  | **No T2DM** | **T2DM** | **p-value** | **No T2DM** | **T2DM** | **p-value** |
| STEMI involving left main coronary artery, n(%) | 262(0.55) | 103(0.66) | 0.281 | 102(0.63) | 32(0.46) | 0.795 |
| STEMI involving left anterior descending coronary artery, n(%) | 6463(13.56) | 1859(11.98) | <0.001 | 1991(12.20) | 689(9.82) | <0.001 |
| STEMI involving other coronary artery of anterior wall, n(%) | 11149(23.40) | 3723(23.99) | <0.001 | 3990(24.46) | 1796(25.60) | <0.001 |
| STEMI involving right coronary artery, n(%) | 7433(15.60) | 2164(13.94) | <0.001 | 2167(13.28) | 771(10.99) | <0.001 |
| STEMI involving other coronary artery of inferior wall, n(%) | 13497(28.33) | 4204(27.09) | <0.001 | 4091(25.08) | 1729(24.64) | <0.001 |
| STEMI involving left circumflex coronary artery, n(%) | 1191(2.50) | 296(1.91) | 0.016 | 274(1.68) | 94(1.34) | 0.456 |
| STEMI involving other sites, n(%) | 2713(5.69) | 843(5.43) | 0.002 | 1079(6.61) | 390(5.56) | 0.008 |
| STEMI of unspecified site, n(%) | 4940(10.37) | 2327(14.99) | 0.001 | 2619(16.05) | 1515(21.59) | 0.018 |
| Age, mean (SD) | 62.87(12.69) | 67.34(11.89) | <0.001 | 71.71(14.37) | 75.87(11.43) | <0.001 |
| CCI, mean (SD) | 0.37(0.28) | 0.61(0.56) | <0.001 | 0.49(0.44) | 0.72(0.65) | <0.001 |
| Obesity, n(%) | 4912(10.31) | 2423(15.61) | <0.001 | 1833(11.24) | 1246(17.76) | <0.001 |
| Hypertension, n(%) | 18681(39.21) | 8698(56.05) | <0.001 | 7708(47.25) | 4242(60.46) | <0.001 |
| Lipid metabolism disorders, n(%) | 19348(40.61) | 8929(57.54) | <0.001 | 6613(40.54) | 3939(56.14) | <0.001 |
| Renal disease, n(%) | 2347(4.93) | 1884(12.14) | <0.001 | 1278(7.83) | 1159(16.52) | <0.001 |
| Atrial fibrillation, n(%) | 4546(9.54) | 1846(11.90) | <0.001 | 2683(16.45) | 1217(17.35) | 0.091 |
| Congestive heart failure, n(%) | 5459(11.46) | 2653(17.10) | <0.001 | 2987(18.31) | 1875(26.72) | <0.001 |
| Peripheral vascular disease, n(%) | 1882(3.95) | 1168(7.53) | <0.001 | 421(2.58) | 310(4.42) | <0.001 |
| Cerebrovascular disease, n(%) | 959(2.01) | 621(4.00) | <0.001 | 536(3.29) | 376(5.36) | <0.001 |
| Dementia, n(%) | 380(0.80) | 215(1.39) | <0.001 | 512(3.14) | 334(4.76) | <0.001 |
| Mechanical ventilation, n(%) | 2845(5.97) | 1094(7.05) | <0.001 | 938(5.75) | 490(6.98) | <0.001 |
| CABG, n(%) | 480(1.01) | 236(1.52) | <0.001 | 92(0.56) | 52(0.74) | 0.113 |
| PCI, n(%) | 31255(65.60) | 9413(60.65) | <0.001 | 8433(51.69) | 3267(46.56) | <0.001 |
| LOHS, median (IQR) | 5.00(4.00) | 5.00(5.00) | 0.89 | 5.00(5.00) | 5.00(5.00) | 0.897 |
| In-hospital mortality, n(%) | 2787(5.85) | 1217(7.84) | <0.001 | 1949(11.95) | 1095(15.61) | <0.001 |

STEMI; ST-elevation myocardial infarction. CCI: Charlson comorbidity index; CABG: Coronary artery bypass graft; PCI: Percutaneous coronary intervention; LOHS: length of hospital stay. The P values for the differences between patients with T2DM and No T2DM were calculated using Student's t-test, or Mann-Whitney test or chi-square tests.
