# Supplementary material for: Are there sex differences in the effect of type 2 diabetes in the incidence and outcomes of myocardial infarction? A matched-pair analysis using hospital discharge data
Source: Cardiovasc Diabetol. 2021 Apr 22;20:81. doi: 10.1186/s12933-021-01273-y (PMC8063379; doi:10.1186/s12933-021-01273-y)
Supplement: Supplementary file 3 — Additional file 3: Table S3. Clinical characteristics, use of therapeutic procedures and hospital outcomes before matching for men and women patients with NSTEMI according to T2DM status. [file 12933_2021_1273_MOESM3_ESM.docx]

TABLE S3. Clinical characteristics, use of therapeutic procedures and hospital outcomes before matching for men and women patients with NSTEMI according to T2DM status.

|  | **MEN** | | | **WOMEN** | | |
| --- | --- | --- | --- | --- | --- | --- |
|  | **No T2DM** | **T2DM** | **p-value** | **No T2DM** | **T2DM** | **p-value** |
| NSTEMI, n(%) | 30309 | 16283 | <0.001 | 13056 | 8204 | <0.001 |
| Age, mean (SD) | 67.20(13.21) | 70.90(11.05) | <0.001 | 74.22(13.41) | 76.94(10.33) | <0.001 |
| CCI, mean (SD) | 0.53(0.44) | 0.86(0.80) | <0.001 | 0.56(0.50) | 0.83(0.80) | <0.001 |
| Obesity, n(%) | 3496(11.53) | 2535(15.57) | <0.001 | 1564(11.98) | 1767(21.54) | <0.001 |
| Hypertension, n(%) | 13730(45.30) | 9291(57.06) | <0.001 | 6612(50.64) | 4695(57.23) | <0.001 |
| Lipid metabolism disorders, n(%) | 14058(46.38) | 10110(62.09) | <0.001 | 5798(44.41) | 4944(60.26) | <0.001 |
| Renal disease, n(%) | 3126(10.31) | 3430(21.06) | <0.001 | 1541(11.80) | 1878(22.89) | <0.001 |
| Atrial fibrillation, n(%) | 4046(13.35) | 2517(15.46) | <0.001 | 2580(19.76) | 1647(20.08) | 0.576 |
| Congestive heart failure, n(%) | 4044(13.34) | 3526(21.65) | <0.001 | 2561(19.62) | 2324(28.33) | <0.001 |
| Peripheral vascular disease, n(%) | 2223(7.33) | 2217(13.62) | <0.001 | 454(3.48) | 548(6.68) | <0.001 |
| Cerebrovascular disease, n(%) | 951(3.14) | 909(5.58) | <0.001 | 479(3.67) | 487(5.94) | <0.001 |
| Dementia, n(%) | 324(1.07) | 215(1.32) | 0.016 | 420(3.22) | 288(3.51) | 0.245 |
| Mechanical ventilation, n(%) | 941(3.10) | 829(5.09) | <0.001 | 397(3.04) | 389(4.74) | <0.001 |
| CABG, n(%) | 1060(3.50) | 739(4.54) | <0.001 | 177(1.36) | 209(2.55) | <0.001 |
| PCI, n(%) | 14545(47.99) | 6982(42.88) | <0.001 | 4276(32.75) | 2715(33.09) | 0.605 |
| LOHS, median (IQR) | 5.00(5.00) | 6.00(6.00) | <0.001 | 6.00(6.00) | 6.00(6.00) | 1.00 |
| In-hospital mortality, n(%) | 1118(3.69) | 790(4.85) | <0.001 | 818(6.27) | 602(7.34) | 0.002 |

NSTEMI; non-ST elevation myocardial infarction CCI: Charlson comorbidity index; CABG: Coronary artery bypass graft; PCI: Percutaneous coronary intervention; LOHS: length of hospital stay. The P values for the differences between patients with T2DM and No T2DM were calculated using Student's t-test, or Mann-Whitney test or chi-square tests.
