# Supplementary material for: Are there sex differences in the effect of type 2 diabetes in the incidence and outcomes of myocardial infarction? A matched-pair analysis using hospital discharge data
Source: Cardiovasc Diabetol. 2021 Apr 22;20:81. doi: 10.1186/s12933-021-01273-y (PMC8063379; doi:10.1186/s12933-021-01273-y)
Supplement: Supplementary file 4 — Additional file 4: Table S4. Logistic regression factors associated with IHM after myocardial infarction among all patients and according to the presence of T2DM to assess the sex differences. [file 12933_2021_1273_MOESM4_ESM.docx]

TABLE S4. Logistic regression factors associated with IHM after myocardial infarction among all patients and according to the presence of T2DM to assess the sex differences.

|  | **No T2DM** | **T2DM** | **BOTH** |
| --- | --- | --- | --- |
| 40-59 years | 1 | 1 | 1 |
| 60-69 years | 1.68(1.38-2.04) | 1.86(1.54-2.24) | 1.76(1.54-2.02) |
| 70-79 years | 2.85(2.38-3.41) | 3.04(2.54-3.62) | 2.94(2.59-3.34) |
| ≥80 years | 6.71(5.61-8.01) | 6.2(5.2-7.38) | 6.43(5.68-7.28) |
| Obesity | 0.72(0.61-0.85) | 0.78(0.7-0.88) | 0.76(0.7-0.84) |
| Hypertension | 0.89(0.81-0.97) | 0.9(0.83-0.98) | 0.9(0.84-0.95) |
| Lipid metabolism disorders | 0.69(0.63-0.75) | 0.75(0.69-0.8) | 0.72(0.68-0.76) |
| Renal diseases | 1.29(1.15-1.44) | 1.29(1.17-1.42) | 1.29(1.2-1.38) |
| Atrial fibrillation | 1.07(0.97-1.17) | 1.16(1.06-1.27) | 1.12(1.05-1.19) |
| Congestive heart failure | 1.86(1.71-2.03) | 1.54(1.43-1.67) | 1.68(1.59-1.78) |
| Peripheral vascular disease | 1.32(1.13-1.54) | 1.17(1.03-1.32) | 1.22(1.11-1.34) |
| Cerebrovascular disease | 1.54(1.32-1.81) | 1.66(1.46-1.89) | 1.61(1.46-1.78) |
| Dementia | 2.4(2.03-2.84) | 2.13(1.82-2.49) | 2.26(2.01-2.53) |
| Mechanical ventilation | 14.13(12.68-15.73) | 10.21(9.24-11.28) | 11.84(11-12.74) |
| CABG | 0.72(0.54-0.96) | 0.49(0.37-0.65) | 0.59(0.48-0.72) |
| PCI | 0.37(0.34-0.41) | 0.41(0.37-0.44) | 0.39(0.37-0.42) |
| STEMI/NSTEMI | 2.56(2.36-2.78) | 2.54(2.35-2.74) | 2.55(2.41-2.7) |
| Diabetes | NA | NA | 1.06(1-1.12) |
| Female sex | 1.11(1.02-1.2) | 1.24(1.15-1.35) | 1.17(1.11-1.24) |
